# The use of evolutionary approaches to understand single cell genomes

**DOI:** 10.3389/fmicb.2015.00191

**Published:** 2015-03-10

**Authors:** Haiwei Luo

**Affiliations:** ^1^Simon F. S. Li Marine Science Laboratory, School of Life Sciences, The Chinese University of Hong KongHong Kong, China

**Keywords:** single cell genomics, phylogenomics, population genomics, homologous recombination, natural selection, genetic drift

## Abstract

The vast majority of environmental bacteria and archaea remain uncultivated, yet their genome sequences are rapidly becoming available through single cell sequencing technologies. Reconstructing metabolism is one common way to make use of genome sequences of ecologically important bacteria, but molecular evolutionary analysis is another approach that, while currently underused, can reveal important insights into the function of these uncultivated microbes in nature. Because genome sequences from single cells are often incomplete, metabolic reconstruction based on genome content can be compromised. However, this problem does not necessarily impede the use of phylogenomic and population genomic approaches that are based on patterns of polymorphisms and substitutions at nucleotide and amino acid sites. These approaches explore how various evolutionary forces act to assemble genetic diversity within and between lineages. In this mini-review, I present examples illustrating the benefits of analyzing single cell genomes using evolutionary approaches.

## Introduction

Single cell genome sequencing technology (single cell isolation, followed by multiple displacement amplification and genome sequencing) has been widely used to unravel the metabolism of uncultivated microbes in the past 5 years, and this trend is expected to continue in that 99% of the environmental microbes remain uncultivated. Analyses of single-cell amplified genomes (SAGs) from the uncharted branches of the tree of life have allowed reconstruction of metabolic potential of ecological key players in various marine and terrestrial environments (Swan et al., 2011; Dupont et al., 2012; Dodsworth et al., 2013; Garcia et al., 2013; Lloyd et al., 2013), extension of our fundamental understanding of biology such as the discovery of reassignment of TGA opal stop codons to glycine (Rinke et al., 2013), and identification of novel natural products for medical applications (Grindberg et al., 2011; Siegl et al., 2011). Moreover, single cells of well described lineages are also sequenced, and comparative analyses have revealed important genomic differences between cultured members and uncultivated counterparts (Swan et al., 2013), and between different habitats such as surface versus deep ocean (Luo et al., 2014c; Thrash et al., 2014).

While definitive assignment of novel metabolic traits to uncultivated lineages is an exciting application of single cell genomics, this effort is sometimes compromised since missing DNA is common in single cell genome amplification with an average of ∼50% loss of the genomic DNA (Rinke et al., 2013; Swan et al., 2013; Luo et al., 2014c). This low recovery rate, however, does not necessarily impede utilization of nucleotide variation information. Recovered DNA through genome amplification is a random sample of the genome, and information gained by analysis of this random sample is thus representative of the genome-scale pattern. Metagenomic fragment recruitment analysis, for instance, assigns the metagenomic DNA fragments to the reference genomes based on a certain sequence similarity cutoff (e.g., 95%), regardless of the genome content (Rusch et al., 2007). Using >50 SAGs from various marine bacterial lineages and global ocean metagenomic reads, the recruitment analysis has led to the finding that global distribution of surface ocean bacterioplankton correlates with temperature and latitude (Swan et al., 2013). When intraspecific genome sequences are available, distinct patterns of single nucleotide polymorphisms (SNPs) of subpopulations may indicate ongoing ecological speciation processes (Shapiro et al., 2012). A recent analysis of ∼90 SAGs in a high-light-adapted ecotype of marine cyanobacterial *Prochlorococcus* showed that a small volume of seawater contains 100s of ecologically distinct subpopulations differing mainly at the SNP level and linked to a limited diversity of flexible genes (Kashtan et al., 2014).

An emerging direction for SAG analysis has taken advantage of molecular evolutionary approaches guided by population genetic theories, with a major goal to understand the role of selection, drift, mutation, and recombination in assembling genetic diversity within and between lineages. While genome content difference is often an important source of information and thus missing genes in SAGs compromise some evolutionary analyses, patterns in polymorphisms and substitutions at single nucleotide sites are most frequently explored by population genetic approaches. For the comparison of more divergent lineages in which nucleotide substitutions are often saturated, the use of sophisticated phylogenetic models correcting for various heterogeneous evolutionary processes is often critical to unravel the ancient diversification processes, and these methods are again based on nucleotide/amino acid substitution models and independent of genome content. In this mini-review, I summarize the studies that make use of the SAG data through evolutionary approaches.

## Homologous Recombination Analysis Using Single Cell Genomes

Homologous recombination is an important evolutionary mechanism shaping the genetic diversity of asexual populations. Understanding homologous recombination rate and pattern requires analyzing closely related sequences varying at the strain level, and this has been done for uncultivated microbes as intraspecific SAGs are becoming available. By analyzing four closely related SAGs of betaproteobacterial *Snodgrassella alvi* and three of gammaproteobacterial *Gilliamella apicola* from the gut of a honey bee, Engel et al. (2014) demonstrated that homologous recombination is common within each of the uncultivated endosymbiotic populations. This conclusion was corroborated by using multiple independent approaches (Engel et al., 2014). First of all, many single gene trees show topological differences from the genome tree, suggestive of frequent recombination though some incongruence may arise from insufficient phylogenetic signal. Next, 13 genes in the *S. alvi* population are associated with unusually large synonymous substitution rate (*d_S_*) and thus are significantly affected by acquisition of divergent alleles through recombination, among which the urease gene cluster might be used to resist acidic stress in the bee gut. The underlying principle is that nucleotide substitutions at synonymous site are largely “invisible” to natural selection. Consequently, the variation of *d_S_* among genes largely reflects stochasticity of mutations and some unusually large values are most likely to arise from recombination. In a third approach, the ratio of probabilities that a given site is altered through recombination versus mutation (r/m) was measured, and the finding of a higher r/m ratio associated with a lineage in *G. apicola* validated the distinct pattern of *d_S_* in this lineage. Finally, 15% of the genes were found to have intragenic recombination (i.e., exchange of small fragments within a gene). In another study of homologous recombination in an uncultivated free-living bacterial lineage LD12 represented by 10 SAGs, Zaremba-Niedzwiedzka et al. (2013) performed the topological comparison between gene tree and genome phylogeny and the r/m measurement, and they concluded that the rate of homologous recombination in the freshwater LD12 bacteria is very low, which is in sharp contrast to their marine relative SAR11 bacteria in which the homologous recombination rate is extremely high.

Single-cell amplified genomes are often incomplete, and hence it is useful to check the completeness requirement of the above approaches. In the r/m measurement and the *d_S_* estimate for homologous recombination, analyses are usually based on the orthologous genes that are present in every member of the taxa under study. In the case of gene tree – genome tree comparison, missing taxa in the gene trees are tolerable, since these missing taxa can be dropped from the genome tree so that the gene tree and genome tree under comparison have the same set of taxa.

## Comparing the Efficiency of Selection Using Single Cell Genomes

**FIGURE 1:**
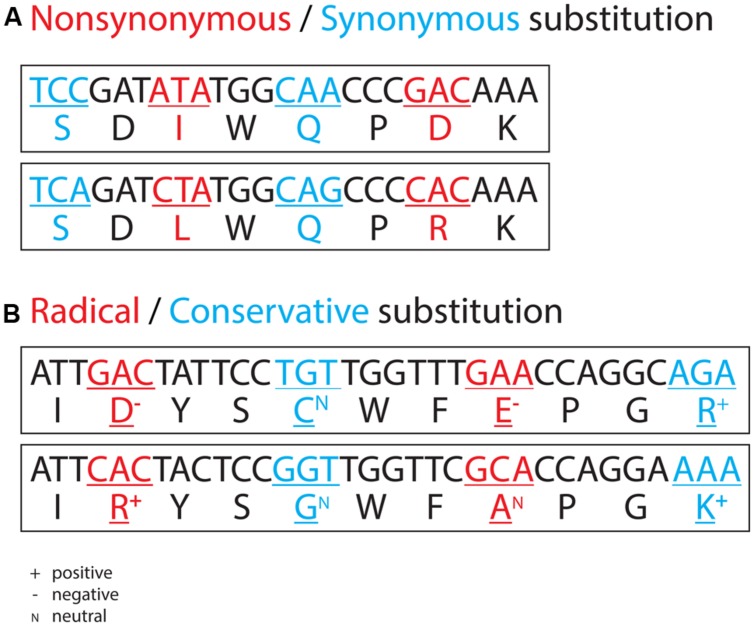
**Examples of non-synonymous and synonymous nucleotide substitutions (A) and examples of radical and conservative non-synonymous nucleotide substitutions (B)**. In the former, a non-synonymous substitution leads to an amino acid replacement, and a synonymous substitution does not. In the latter, the 20 amino acids show different physicochemical properties, such as charge. A radical non-synonymous substitution leads to an amino acid replacement with a concomitant change in charge, and a conservative non-synonymous substitution leads to an amino acid replacement without a change in charge.

Closely related genomes can also be used to compare the efficiency of selection among lineages. Efficiency of selection largely determines whether mildly favorable mutations can be effectively spread and mildly deleterious mutations can be effectively eliminated, and thus determines the adaptive potential of a population. It is often denoted by the ratio (ω) of the number of non-synonymous substitutions (**Figure 1A**) per non-synonymous site (*d*_N_) to the number of synonymous substitutions (**Figure 1A**) per synonymous site (*d_S_*). Theory predicts that a genome-wide inflation of ω is a result of reduced efficiency of selection (Ohta, 1992). While a largely uncultivated lineage (represented by the strain HTCC2255 and a closely related SAG) is more abundant in the oceanic waters and thus seemingly more successful, most cultured lineages are under more efficient selection (with significantly lower ω) and thus have a greater capability to adapt in a changing ocean (Luo et al., 2014a). It is important to note that there is a considerable difference in genomic G+C content between this largely uncultivated lineage (37% in both genomes) and all cultured ones (60 ± 4%), and evolutionary models of nucleotide substitution that do not take into account the base frequency bias, including the frequently used Nei and Gojobori (1986) method, will lead to highly biased estimates of *d_S_* and hence erroneous inference of selective pressure on the functional genes (Luo and Hughes, 2012) and selection efficiency of the populations. As single cells with G+C-poor genomes are prevalent in marine planktonic bacteria and obligate intracellular bacteria (McCutcheon and Moran, 2012; Swan et al., 2013), it is strongly recommended to use an appropriate model for *d_S_* calculation, which can be determined using the KaKs_Calculator software (Zhang et al., 2006).

Often, closely related genomes are not available, as is the case of an uncultivated Roseobacter lineage (named SAG-O19) exclusively represented by three divergently related single cells (Luo et al., 2014b). In this case, nucleotide substitutions at synonymous site are saturated, and therefore approaches involving the *d*_S_ measurement fail. For such divergent taxa, non-synonymous substitutions usually remain informative. A non-synonymous nucleotide substitution leads to an amino acid replacement, which can be either radical or conservative depending on the difference in physicochemical property (e.g., charge, polarity, volume) between the two amino acids in replacement (**Figure 1B**). Further, rates of these two types of non-synonymous substitutions, that is, the number of radical non-synonymous substitutions per radical non-synonymous site (*d*_R_) and the number of conservative non-synonymous substitutions per conservative non-synonymous site (*d*_C_), are measurable (Hughes et al., 1990; Zhang, 2000). Theory predicts that inefficient selection leads to a genome-wide inflation of the ratio of *d*_R_ to *d*_C_. However, the available computer program (Zhang, 2000) cannot account for the possible effect of biased nucleotide content on the measurement of *d*_R_ and *d*_C_, leading to equivocal interpretations of the pattern derived from the analyses of genomes displaying considerable variability in G+C content. For instance, the genomic G+C content of the uncultivated SAG-O19 lineage (39 ± 1%) is substantially lower than that of cultured Roseobacters (60 ± 4%). Although an inflated genome-wide *d*_R_ to *d*_C_ ratio was found in SAG-O19 compared to any cultured Roseobacter lineage, ascription of this inflation to inefficient selection on the SAG-O19 clade requires additional evidence (Luo et al., 2014b). The two methods (i.e., *d*_N_/*d*_S_, *d*_R_/*d*_C_) presented here for comparing selection efficiency both use a statistical approach (paired *t*-test or sign test) to compare the mean or median values of the genome-wide orthologous genes among multiple lineages. These statistical approaches allow missing data, thus making SAGs suitable for the analyses of selection efficiency.

## Sequencing Error in Single Cell Genomics

Variation at single nucleotide site could also be generated by sequencing errors. Indeed, using benchmark SAGs, an error frequency of 5–200 bases per Mb has been estimated (Rodrigue et al., 2009; Nurk et al., 2013; Zaremba-Niedzwiedzka et al., 2013; Kashtan et al., 2014). However, this error rate is often two orders of magnitude smaller than the amount of polymorphisms contained in the data, and thus this low error rate is considered having little impact on the analyses of the available intraspecific SAGs (Zaremba-Niedzwiedzka et al., 2013; Kashtan et al., 2014). Moreover, errors are expected to be randomly distributed along the genome and not to differentiate between synonymous and non-synonymous sites, but the observed single nucleotide variations are often clustered (Kashtan et al., 2014) and the nucleotide substitution rate at synonymous site exceeds that at non-synonymous site by a factor of 10 (Luo et al., 2014a). These analyses strongly suggest that the single nucleotide variations are primarily generated through biological processes.

## Evolutionary Changes of Genome Content

Gene flux analysis has been used in a number of microbial genome evolutionary studies. It adds significant insights into evolutionary changes of genome content through reconstructing the number of gene gains and losses during the history of a group of related organisms. It, however, becomes less applicable to partial single cell genomes, since missing genes can be either truly absent, or simply not amplified and sequenced. In their gene flux analysis of the marine SAR11 lineages and its uncultivated freshwater relative LD12 lineage represented by 10 SAGs, Zaremba-Niedzwiedzka et al. (2013) treated the LD12 lineage as a single phylogenetic branch and used the total set of genes in all 10 SAGs to represent the genome content of LD12. This approach is useful when the uncultivated microbes of interest comprise a monomorphic lineage with a limited size of pan-genome and the sequenced members have already captured the diversity of the natural populations, both of which were demonstrated in their analyses of LD12 (Zaremba-Niedzwiedzka et al., 2013).

In some lineages, ecological subpopulations associated with distinct habitats co-occur with phylogenetic differentiation, and identification of a few ecologically relevant genes that are specific to each subpopulation may be linked to the adaptive mechanism in these habitats. One example is the rarely cultivated marine Thaumarchaeota. Single marker gene (e.g., 16S ribosomal RNA) analyses consistently showed that they fall into two phylogenetically distinct groups corresponding to shallow- and deep-water clades. This depth distribution has been hypothesized to be related to photoinhibition (Mincer et al., 2007). Phylogenomic analysis of 46 SAGs validated this phylogenetic structure. Interestingly, a DNA photolyase gene responsible for repairing ultraviolate-induced DNA damage and two catalase genes resisting oxidative stress possibly caused by photo-oxidation were exclusively found in the shallow-water clade, which is represented by only four single cells (Luo et al., 2014c). Although genomes of these 46 single cells are only partially recovered (32 ± 12%), the observation that none of the 42 deep-water SAGs contain the photolyase and catalase genes strongly suggests that these genes are truly absent from the deep-water clade members. Therefore, the exclusive occurrence of these genes in members of the shallow-water clade is an adaptive mechanism to reduce light-induced damages in illuminated waters (Luo et al., 2014c).

## Phylogenomic Analyses of Single Cells

A few SAGs are associated with certain uncharted branches of the tree of life, and phylogenetic placement based on a concatenated sequence of multiple proteins is a common practice. For instance, Rinke et al. (2013) recently sequenced 201 SAGs covering 29 major undescribed deeply branching lineages, and their phylogenomic analysis along with other known major lineages validated the occurrence of several superphyla that were previously proposed based on rRNA gene sequences. They further proposed new hypotheses of evolutionary positions of a few lineages that challenged well-accepted concepts (Rinke et al., 2013).

On the other hand, many SAGs are members of well-studied lineages with cultured isolates, but genomic traits of these SAGs do not necessarily match their cultivated relatives. This has been well documented in various surface ocean bacterial lineages (Swan et al., 2013). Among the several systematic differences between cultured and uncultivated cells, heterogeneity in amino acid frequency and G+C content are known to be a potential source of systematic errors in molecular phylogenetic reconstruction. If not appropriately accounted for, it may result in statistically supported artifact by clustering compositionally similar sequences that do not have biological relatedness, which has been illustrated in a number of phylogenetic studies (Galtier and Gouy, 1995; Herbeck et al., 2005; Foster et al., 2009; Nesnidal et al., 2010; Morgan et al., 2013; Cox et al., 2014; Li et al., 2014; Liu et al., 2014; Luo, 2015).

**FIGURE 2:**
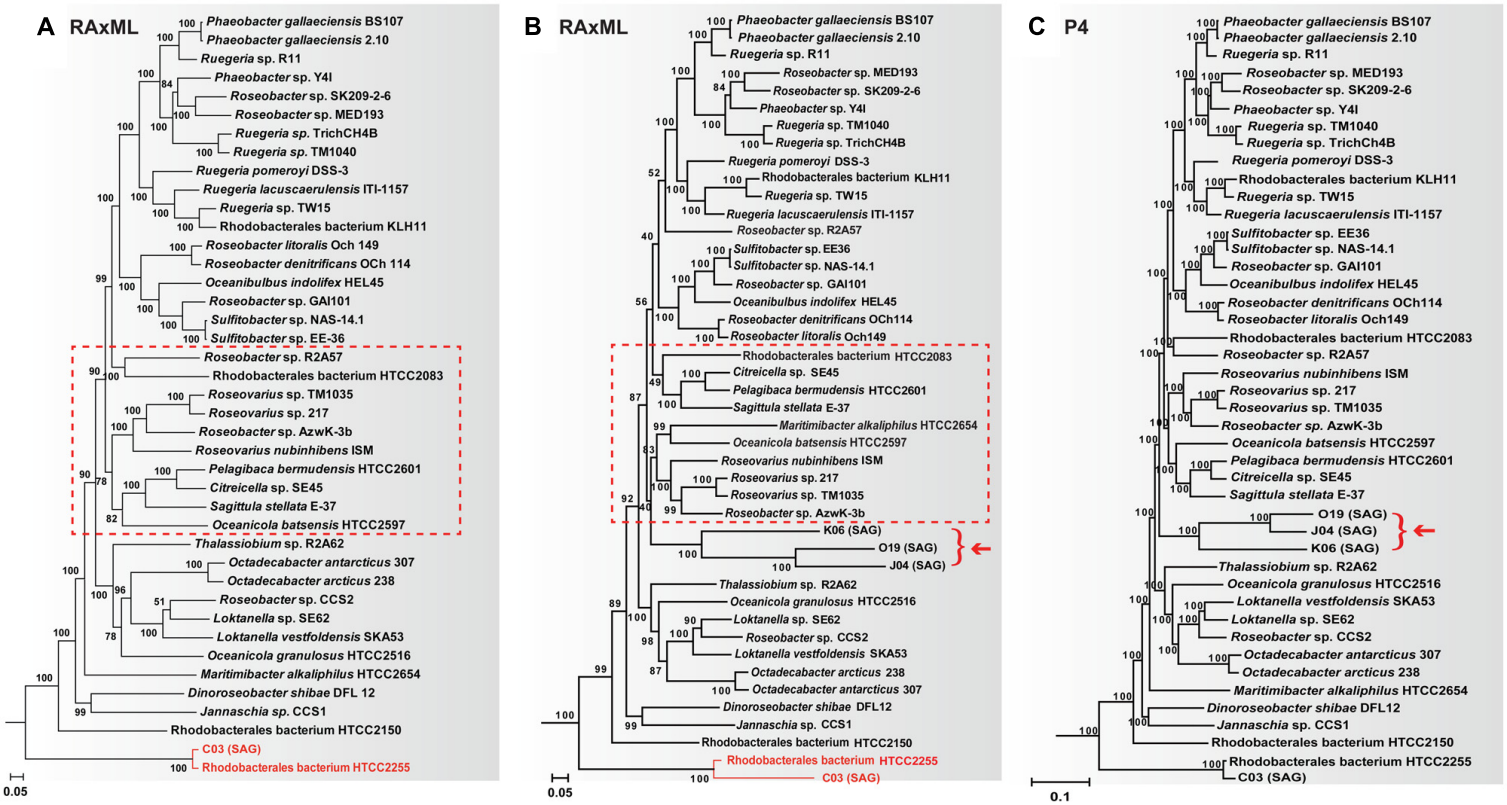
**The effect of adding the SAG-O19 clade (represented by K06, O19, J04, and pointed by an arrow) on the resulting Roseobacter phylogeny.** All trees are based on a concatenated sequence of 52 single-copy orthologous proteins shared by every member of the Roseobacter clade. **(A)** RAxML tree without SAG-O19; **(B)** RAxML tree with SAG-O19; **(C)** P4 tree with SAG-O19 using the NDCH model. Major differences between **(A,B)** are highlighted. Figure adapted from (Luo et al., 2014b).

One bacterial group containing both cultured and single cell lineages with a substantial compositional difference is the marine Roseobacter clade. Roseobacters are a dominant bacterial group in global oceans, playing a significant role in marine carbon and sulfur cycles (Luo and Moran, 2014). Many cultured Roseobacters have genomic G+C content >60%, while members of an uncultivated SAG-O19 clade consistently have this trait <40% (Luo et al., 2014b). Considerable among-taxa compositional difference was also found in their amino acid sequences (Swan et al., 2013). Based on concatenated sequences consisting of 52 single-copy orthologous proteins shared by all Roseobacters, a significantly biased tree was produced using the maximum likelihood RAxML software (Stamatakis, 2014), in which two genomes (HTCC2255, SCGC AAA076-C03) sharing an identical 16S rRNA gene sequence display a huge difference in their branch lengths (**Figure 2B**; Luo et al., 2014b). This branch length difference disappeared when the SAG-O19 clade was not included in the RAxML analysis (**Figure 2A**) or when a node-discrete composition heterogeneity (NDCH) model in the P4 software (Foster, 2004) was employed (**Figure 2C**; Luo et al., 2014b). Moreover, the complete P4 tree and the reduced RAxML tree have identical branching order in regards to the cultured Roseobacters (Figures 2A,C), but inclusion of the SAG-O19 clade in the RAxML analysis breaks up a few established clusters (**Figure 2B**; Luo et al., 2014b).

Long-branch attraction (LBA) is another long-standing issue encountered in various phylogenetic analyses, leading to artificial grouping of lineages with accelerated and parallel changes of molecular sequences. Since genome streamlining is often associated with long branches (Viklund et al., 2012, 2013; Luo et al., 2014b), and since genome streamlining is prevalent in surface ocean SAGs (Swan et al., 2013), LBA is likely to be an important issue that, though currently unrecognized in SAG analyses, deserves attention in future phylogenetic analyses of surface ocean SAGs.

## Concluding Remarks

Single cell genome sequencing has been extensively used to reconstruct the metabolism of uncultivated microbes, but its potential in molecular evolutionary analyses has not been fully explored. SAG sequences differentiate strains at single nucleotide site, and such intra- and inter-specific variations are the key information that various population genetic approaches look for. It is useful to compare the level of polymorphism contained in the population to the estimated amount of sequencing errors, since single cell genome sequencing may have a greater error rate compared to conventional genome sequencing. The major limitation of using SAGs for evolutionary analyses is its incompleteness, which limits gene flux analysis and ancestral genome reconstruction. In addition, theoretical studies demonstrated that missing a significant amount of genes in the concatenated sequences may be detrimental to phylogenetic inferences (Lemmon et al., 2009; Wiens and Morrill, 2011; Roure et al., 2013). More simulation and empirical studies are desirable to understand how missing genes interact with different phylogenetic models, with a goal to come up with an easy-to-follow guideline for taxa and character sampling with various recovery rates of SAGs. Despite these challenges, SAGs have proven to be an exceptional source of genetic data for molecular evolutionary analyses, and the more widespread use of population genomic and phylogenomic approaches guarantees to improve our understandings of the functional roles of uncultivated microbes in nature and how genetic diversity evolved and is maintained in natural populations.

## Conflict of Interest Statement

The Editor Brandon K. Swan declares that, despite having collaborated with author Haiwei Luo, the review process was handled objectively and no conflict of interest exists. The author declares that the research was conducted in the absence of any commercial or financial relationships that could be construed as a potential conflict of interest.
